# Acute invasive fungal sinusitis with orbital tip syndrome in patients on long-term use of ruxolitinib: a case report

**DOI:** 10.1186/s13256-024-04486-3

**Published:** 2024-04-05

**Authors:** Zhiyuan Tang, Zhaohui Shi

**Affiliations:** 1 Department of Otolaryngology Head and Neck Surgery, Shenzhen University General Hospital, Shenzhen, 518055 China; 2 Department of Otorhinolaryngology-Head and Neck Surgery, Department of Allergy, Naso-Orbital-Maxilla and Skull Base Center, The Third Affiliated Hospital of Sun Yat-sen University, Guangzhou, 510630 Guangdong China; 3 Departmentof Otolaryngology, Shenzhen Longgang Otolaryngology Hospital & Shenzhen Otolaryngology Research Institute, Shenzhen, 518172 Guangdong China

**Keywords:** Acute invasive fungal sinusitis, Orbital tip syndrome, Ruxolitinib

## Abstract

**Introduction:**

A long-term ruxolitinib-treated patient with primary myelofibrosis, who was co-infected with aspergillosis infection during a short period, developed acute invasive fungal sinusitis with consequent orbit apex syndrome. This may be the first reported case in the world.

This is a 75-year-old Chinese man; the patient was admitted with 2-month history of headache accompanied by numbness and 8-day history of vision loss. The preliminary clinical diagnoses were suspected acute invasive fungal sinusitis or adenoid cystic carcinoma. We performed endoscopic debridement and antifungal therapy. About 90 days after surgery, magnetic resonance imaging revealed no recurrence of pathological tissue.

**Conclusion:**

One of the bases for the occurrence of invasive fungal sinusitis may be the patient’s long-term use of ruxolitinib for essential thrombocythemia. Some patients with invasive fungal sinuses have atypical nasal symptoms and are referred to the corresponding departments with eye and headache as the first symptoms. It is suggested that enhanced magnetic resonance imaging should be performed at an early stage. Surgical treatment in combination with antifungal and enhanced immunotherapy can effectively prevent the spread of infection and reduce the risk of death.

## Introduction

### Case presentation

Ruxolitinib is a novel potent biologic agent that inhibits Janus kinase 1 (JAK1) and Janus kinase 2 (JAK2), reserved for patients with myeloproliferative diseases [1]. Although it is effective and improves survival in such patients, the immunosuppressive agent may increase the risk of acquiring opportunistic infections. However, very little data are available regarding the infectious complications while on this agent. Previous studies have reported bacteria, *Mycobacterium tuberculosis*, *Cryptococcus neoformans*, *Pneumocystis jirovecii*, herpes simplex, and varicella-zoster virus to be etiologic agents that had been isolated from patients receiving ruxolitinib [2]. Here, we report a long-term ruxolitinib-treated patient with primary myelofibrosis, who was coinfected with aspergillosis infection during a short period and developed acute invasive fungal sinusitis with consequent orbit apex syndrome.

### Patient information

This is a 75-year-old Chinese man; the patient was admitted with a 2 month history of headache accompanied by numbness and an 8 day history of vision loss.

### Timeline

From November 2021 onward, the patient experienced right-sided toothache and sought treatment at other hospitals. Computed tomography (CT) examination suggested possible temporomandibular joint (TMJ) disorder; however, conservative management proved ineffective as the patient developed persistent swelling pain and unbearable temporal pain. Subsequently, he was admitted to the pain department where he received a diagnosis of TMJ pain and trigeminal neuralgia.

After conservative treatment proved ineffective and the patient experienced persistent head and facial numbness, as well as gradually worsening head and facial pain that became unbearable, they sought medical attention at multiple hospitals but were unable to receive a proper diagnosis or effective symptomatic treatment. In mid-November, the above-mentioned symptoms significantly exacerbated, accompanied by intermittent confusion. The diagnosis was great occipital neuralgia in the pain department for localized treatment followed by minimally invasive surgery.

Although head and facial pain were substantially alleviated, numbness gradually intensified while swelling, protrusion, and decreased vision manifested in the right eye. In early January 2022, he was admitted to the ophthalmology hospital owing to a decrease in visual acuity in his right eye. The orbital CT scan revealed bilateral proptosis without evidence of extraocular muscle thickening. Sinus magnetic resonance imaging (MRI) demonstrated inflammation in the right maxillary, bilateral ethmoid, and sphenoidal sinuses with increased orbital fat.

The right eye experienced a loss of light sensation and subsequently became blind within a few days. He was admitted with the diagnosis of “a space-occupying lesion in the infratemporal fossa and skull base: suspected acute invasive fungal sinusitis and adenoid cystic carcinoma" and “orbital apex syndrome (right).” The patient was diagnosed with thrombocythemia in 2006 and was initially treated with hydroxyurea for 10 years. Since 2017, he had received ruxolitinib for the treatment of myelofibrosis for 4 years. He had received long term blood transfusions with diabetes denial.

### Clinical findings

**Fig. 1:**
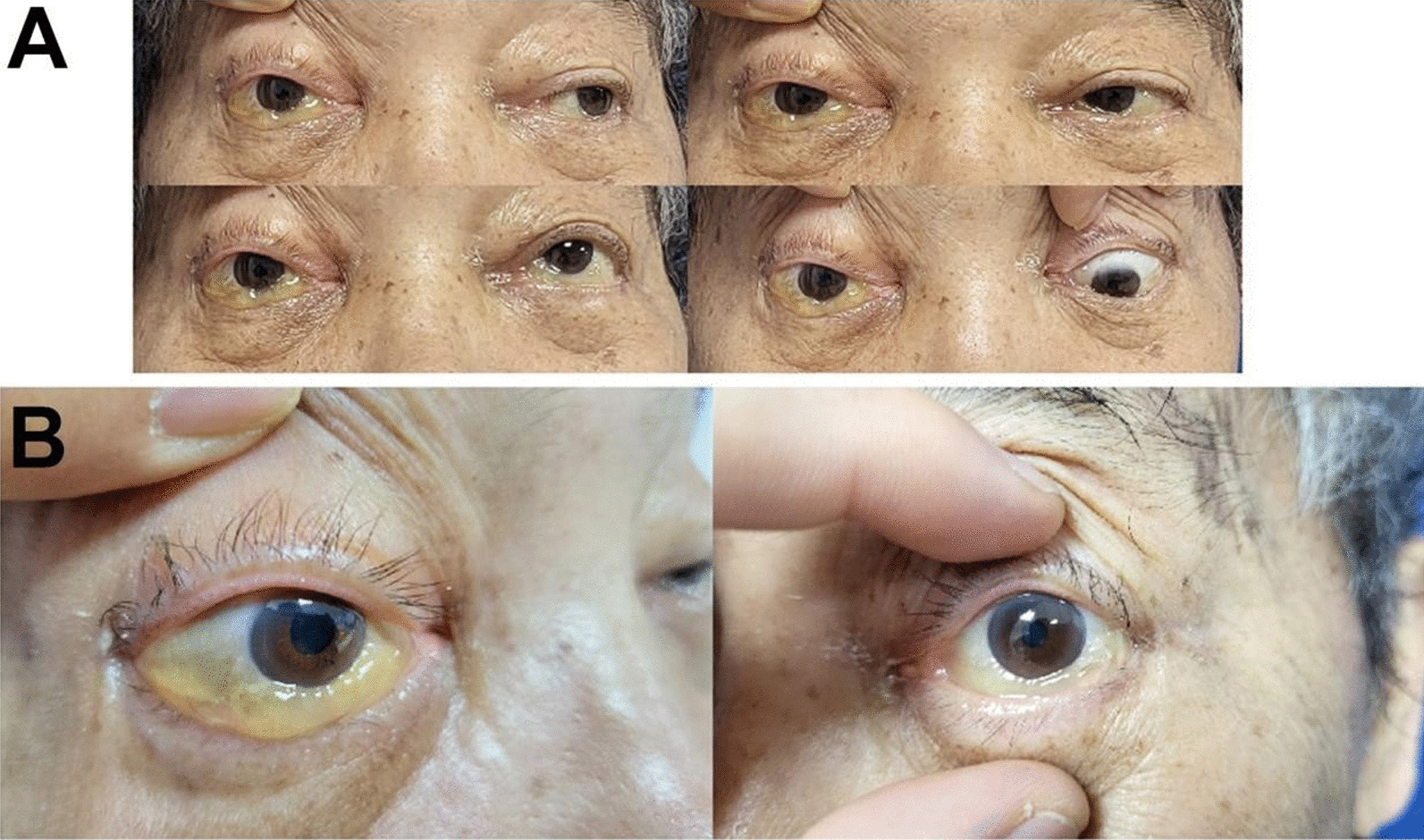
Eye examination of patients before surgery

Physical examination revealed stable vital signs, including a temperature of 36.5 °C and a normal level of consciousness. The right eyelid was droopy, swollen, and could not elevate properly, accompanied by a bulging eyeball, conjunctival chemosis, and a transparent cornea. The pupil of the right eye was dilated by 5 mm, and the right direct and indirect reflexes were lost. The pupil size of the left eye was 3 mm, with normal reflexes. The right middle face and upper lip were numb, while the sensation from the tongue and in the lower lip was normal (Fig. 1). Examination of the nose revealed a pale nasal mucosa, swelling of both inferior turbinates, and no secretion or lump. An internal endoscopic examination of the nose showed a deviated nasal septum, dilation of vessels, swelling of the mucosa in the nasopharynx, and a lump with a smooth surface arising from the roof and posterior wall of the nasopharynx.

**Fig. 2:**
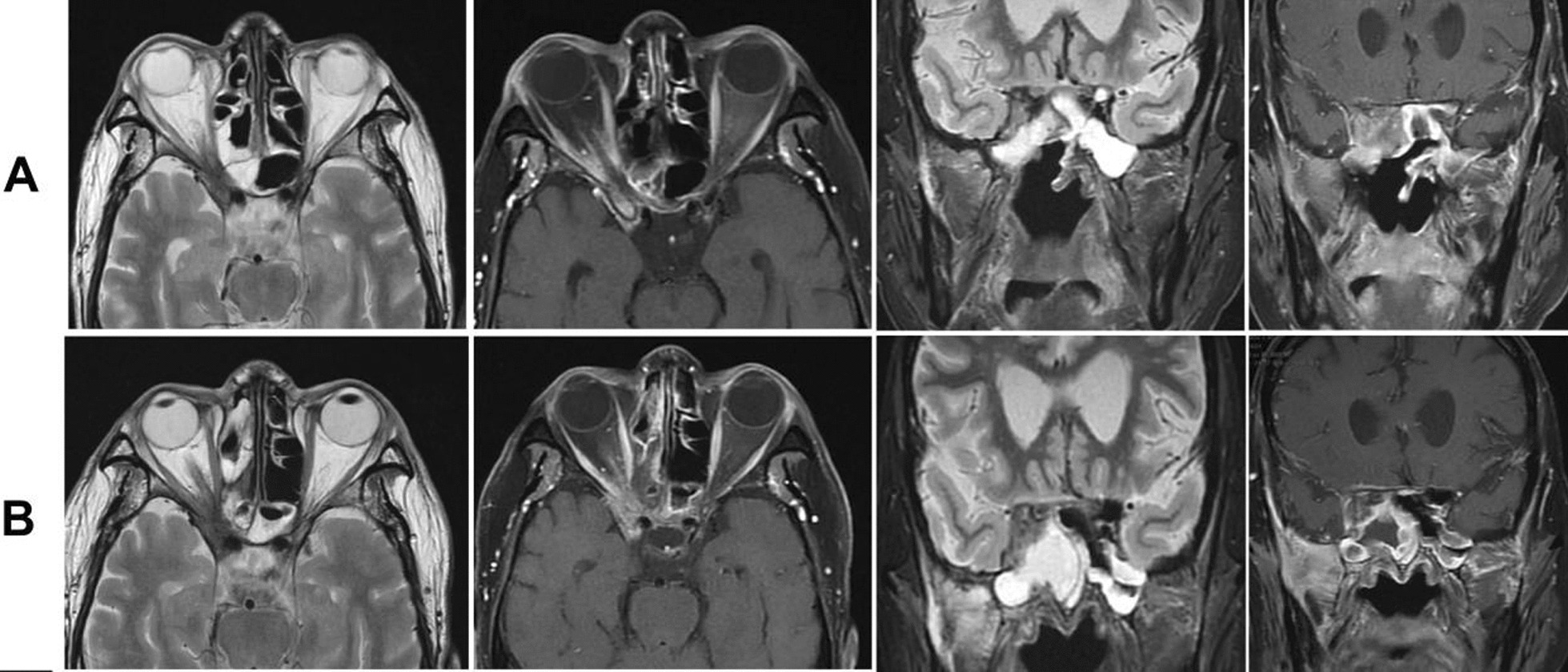
Comparison of magnetic resonance images of the sinuses before and after treatment: **A** 3 months after treatment; **B** before treatment

Magnetic resonance imaging (MRI) scan of the sinuses indicated a right maxillary and sphenoid lesion extending to the pterygopalatine fossa, orbital apex, infratemporal fossa, extraconal fat, right cavernous sinus, and meninges situated in the bilateral anterior and middle skull base (Fig. 2B). We suspected neoplasms (adenoid cystic carcinoma) and chronic invasive fungal sinusitis. The random blood glucose level was between 7.42 and 9.56 mmol/L. Urinalysis revealed that the amount of glucose in urine was more than 1 mmol/L. Glycated hemoglobin was more than 6.0%. Combined with the medical history, the preliminary clinical diagnoses were as follows: (1) a space-occupying lesion in the infratemporal fossa and skull base: suspected acute invasive fungal sinusitis or adenoid cystic carcinoma; (2) orbital apex syndrome (right); (3) severe anemia; (4) myelofibrosis; and (5) diabetes.

### Therapeutic and intervention

The preoperative evaluation was done before undergoing endoscopic sinus surgery. The endoscopic transethmoidal sphenoidotomy with maxillary antrostomy was performed for surgical excision of lesions, which were suspected to be located on the right side of the pterygopalatine fossa, infratemporal fossa, skull base, orbital apex, or cavernous sinus, accompanied by the partial removal of the right middle turbinate, right medial orbital wall, right maxillary bone, and right inferior turbinate bone.

During surgery, fungal balls were seen in the maxillary sinus with evident edema of the sinus mucosa. Bone fragments of the maxillary sinus posterior wall and the lateral orbital wall were broken off. Necrosis was seen in the lateral pterygoid muscle and numerous soft tissue masses, and purulent discharge was visible in the tissue spaces. The necrotic bone of the pterygopalatine fossa eroded through the inferior orbital fissure and extended to the orbital floor and orbital apex. Apart from necrosis of the maxillary nerve, the lesion led to muscle necrosis and the extension of the fracture from the inferior and medial walls of the orbit to the orbital apex, where necrosis of the muscle fascia and orbital contents was partially observable.

**Fig. 3:**
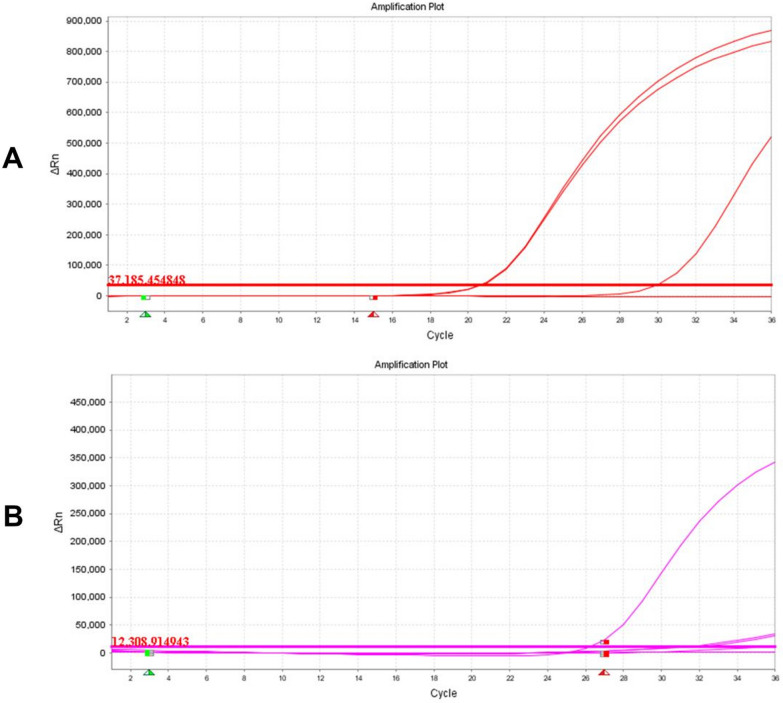
DNA of pan *Aspergillus* detected in diseased tissue: **A** pan *Aspergillus* detection curve; **B** internal extraction control DNA detection curve

Through the right optic canal, a pale and necrotic optic nerve was found. Combined with intraoperative and postoperative fungal antigen detection in blood and tissue (Fig. 3). Postoperatively, the patient was treated with ceftriaxone at 2.0 g/day, voriconazole at 400 mg/day (200 mg twice a day) on the first day [followed by 200 mg/day (100 mg twice a day)], and human immunoglobulins at 10 g/day. At 5 days after surgery, he was referred to the division of hematology to continue antifungal treatments for myelofibrosis.

### Follow-up and outcomes

**Fig. 4:**
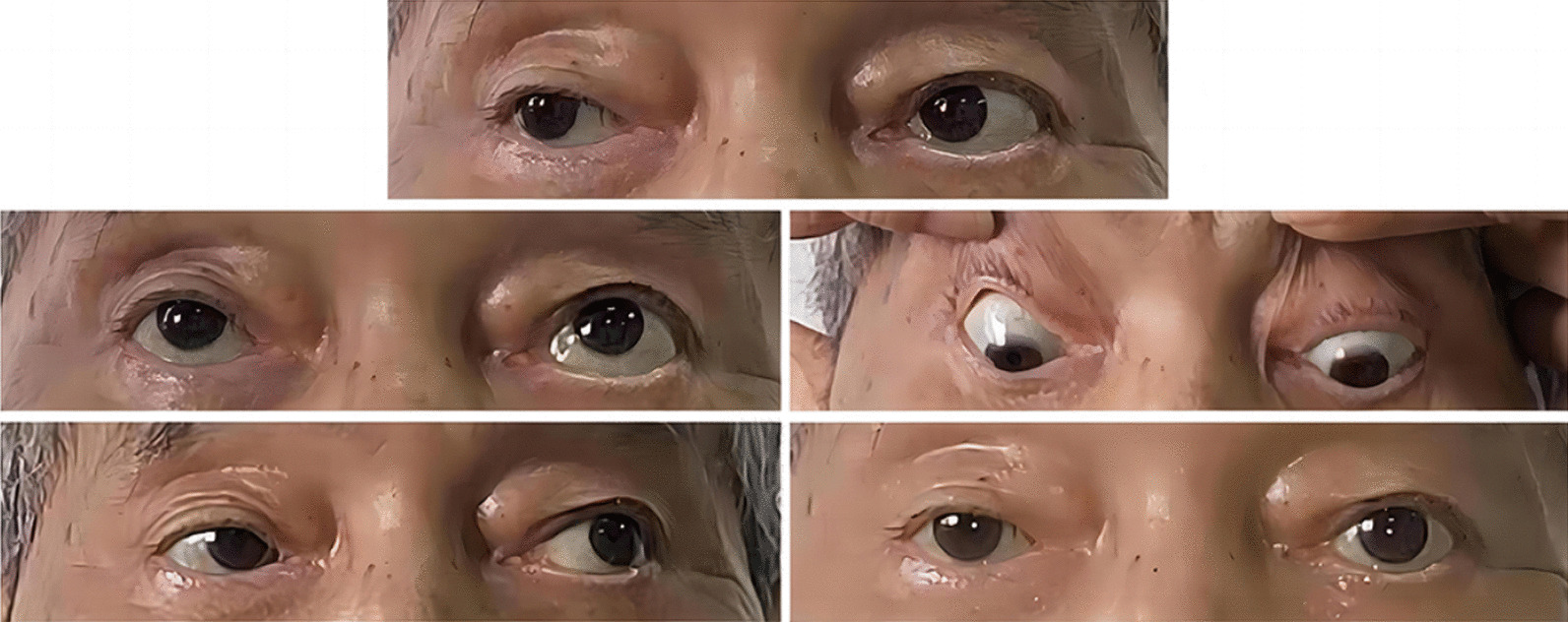
Eye examination of patients about 90 days after surgery

Oral voriconazole was continued after surgery. About 90 days after surgery, the MRI revealed no recurrence of pathological tissue (Fig. 2A). The upper eyelid of the affected eye was able to be fully elevated, and conjunctival edema resolved; however, regrettably, the patient’s right pupil dilated by 5 mm, and the elimination of both direct and indirect light reflections was observed, resulting in complete loss of vision (Fig. 4). This may be attributed to the rapid progression of acute invasive fungal rhinosinusitis resulting in misdiagnosis during the early stages and irreversible optic nerve damage.

### Discussion and conclusion

Fungi produce large numbers of asexual spores called conidia that are released into the air. Humans inhale hundreds to thousands of these infectious propagules every day. In immunocompetent individuals, the cooperation of the respiratory epithelium, lung-resident macrophages, and recruited neutrophils and monocytes clear conidia efficiently. However, in immunocompromised states, it can lead to invasive fungal infections. It has been reported that in patients with myelofibrosis, the hazard ratio of bacterial infections was 1.9 times as high as that of normal people, viral infections were 2.1 times as high, and fungal infections were the highest at about 2.9 times [3].

Myelofibrosis, belonging to a group of diseases called myeloproliferative disorders, is a type of blood cancer characterized by chronic hematologic malignancies [3, 4]. The deregulation of the JAK/signal transducers and activators of transcription pathway leads to myelofibrosis that can be treated by ruxolitinib. However, a growing body of clinical data suggests that ruxolitinib exerts potent anti-inflammatory and immunosuppressive effects [5], interferes with the signaling of several cytokines and growth factors associated with the immune system, and decreases levels of proinflammatory cytokines in patients with myelofibrosis [6]. Furthermore, several studies indicate that ruxolitinib is associated with Treg cell-mediated downregulation, impaired dendritic cell function [7–10], and reduction in natural killer (NK) cell numbers. Although the exact relationship between its effects on immune function and the incidence of infection remains to be elucidated, these immunosuppressive mechanisms may contribute to the increased risk of infection in patients treated with ruxolitinib. A study conducted by Polverelli *et al*. of 507 patients with myelofibrosis showed a 15% correlation between the use of ruxolitinib and aspergillosis as well as other severe infections [11]. Nevertheless, no cases of acute invasive fungal sinusitis owing to aspergillosis in patients with myelofibrosis with ruxolitinib treatment have been reported in literature. 

The patient in this case had a history of myelofibrosis with long-term ruxolitinib treatment, which may have been the main cause of acute invasive fungal sinusitis.

Orbital apex syndrome is a rare ocular complication usually resulting from infection, inflammation, neoplasms, and vascularity, which can lead to damage to the superior orbital fissure and optic canal, resulting in optic nerve dysfunction. Immunocompromised patients, such as those with diabetes or receiving immunosuppressive drugs, are susceptible to fungal infections. Orbital apex syndrome owing to aspergillosis has been reported in an immunocompromised patient with persistent methylprednisolone treatment during chemotherapy for metastatic colorectal cancer [12, 13]. However, no cases of orbital apex syndrome caused by aspergillosis have been reported in patients undergoing long-term treatment with ruxolitinib.

The patient typically presented with proptosis, rapid vision loss leading to eventual blindness, visual fixation, a 5 mm dilated pupil in the right eye, loss of direct and indirect light reflexes, and droopy eyelids. On the other hand, the head MRI identified multiple foci on both sides of the face and cavernous sinuses, as well as the right side of the sphenoid sinus, ethmoid sinus, temporal lobe, and retrobulbar space. Infectious lesions and abscesses were diagnosed on the basis of the former comprehensive investigation. We found that the extension of fungal infection from the sinuses to the orbital apex was the pivotal reason for orbital apex syndrome in this case, with the disease progressing aggressively.

In this case, aspergillosis was generally caused by *Aspergillus niger*, *Aspergillus fumigatus*, and *Aspergillus flavus*. The lesion extended to the orbital apex resulting in orbital apex syndrome, thus making the condition complex and difficult to treat. In terms of treatment, we suggested that early surgical intervention was of paramount importance. Prompt surgical care after the patient’s admission and investigations, as well as timely removal of fungal masses and necrotic tissue from the nasal cavity and sinuses, is the key to treatment, which can effectively limit the further extension of the infection. Early empirical antifungal treatment and debridement can potentially reduce morbidity and mortality [14]. Antifungal agents that have been reported to be used successfully include itraconazole and fluconazole to treat aspergillosis [15]. However, patients with myelofibrosis have associated autoimmunity and hypoproteinemia, which contribute to aspergillosis. Hence, simultaneous enhancement of patient immunity in treatment with combination antifungal therapy of voriconazole with human immunoglobulins significantly strengthens the anti-*Aspergillus* effects.

In spite of timely surgery and 6 weeks of antifungal treatment, it is still difficult to predict prognosis. After discharge, computed tomography (CT) scans should be performed every 3–4 months, as well as nasal endoscopy every 2–3 months, to check for local recurrence or recurrence of lesions in the eye, brain, and adjacent organs [16].

From the experience of this case, we have learned that we should carefully evaluate the possible potential risk of infection before ruxolitinib initiation. Currently, this is the first case of aspergillosis with acute invasive fungal sinusitis in a patient undergoing treatment with ruxolitinib for primary myelofibrosis. This case highlights the possibility of acute invasive fungal sinusitis with more serious complications in immunosuppressed patients, especially those receiving long-term ruxolitinib treatment.

## Data Availability

All data generated or analyzed during this study are included in this published article.
